# Whole-genome association study searching for QTL for *Aeromonas salmonicida* resistance in rainbow trout

**DOI:** 10.1038/s41598-021-97437-7

**Published:** 2021-09-08

**Authors:** Moonika H. Marana, Asma M. Karami, Jørgen Ødegård, Shaozhi Zuo, Rzgar M. Jaafar, Heidi Mathiessen, Louise von Gersdorff Jørgensen, Per W. Kania, Inger Dalsgaard, Torben Nielsen, Kurt Buchmann

**Affiliations:** 1 Laboratory of Aquatic Pathobiology, Department of Veterinary and Animal Sciences, Faculty of Health and Medical Sciences, University of Copenhagen, Frederiksberg C., Copenhagen, Denmark; 2 Aquagen, Trondheim, Norway; 3 Institute of Aquatic Resources, Technical University of Denmark, Kgs. Lyngby, Denmark; 4 Aquasearch Ova ApS, Jelling, Denmark

**Keywords:** Gene expression analysis, Genome-wide association studies, Bacterial infection, Gene regulation in immune cells

## Abstract

*Aeromonas salmonicid*a subsp. *salmonicida*, the causative agent of furunculosis, has extensive negative effects on wild and farmed salmonids worldwide. Vaccination induces some protection under certain conditions but disease outbreaks occur even in vaccinated fish. Therefore, alternative disease control approaches are required to ensure the sustainable expansion of rainbow trout aquaculture. Selective breeding can be applied to enhance host resistance to pathogens. The present work used genome-wide association study (GWAS) to identify quantitative trait loci (QTL) associated with *A. salmonicida* resistance in rainbow trout. A total 798 rainbow trout exposed to *A. salmonicida* by bath challenge revealed 614 susceptible and 138 resistant fish. Genotyping was conducted using the 57 K single nucleotide polymorphism (SNP) array and the GWAS was performed for survival and time to death phenotypes. We identified a QTL on chromosome 16 and located positional candidate genes in the proximity of the most significant SNPs. In addition, samples from exposed fish were examined for expression of 24 immune-relevant genes indicating a systematic immune response to the infection. The present work demonstrated that resistance to *A. salmonicida* is moderately heritable with oligogenic architecture. These result will be useful for the future breeding programs for improving the natural resistance of rainbow trout against furunculosis.

## Introduction

*Aeromonas salmonicida* subsp. *salmonicida* is a ubiquitous Gram-negative bacterium, causing furunculosis in wild and captive salmonids in fresh- and saltwater worldwide^1^. Its high pathogenic potential is a serious threat in aquaculture, causing significant morbidity and mortality in intensive salmonid rearing systems^2^. Elevated water temperatures are beneficial for the growth of *A. salmonicida* and high stocking densities in aquaculture increase transmission of bacteria between fish^3^. Climate changes may increase the prevalence of furunculosis in both wild^4^ and aquacultured fish^5^, and with the expanding aquaculture activities and increasing water temperatures no self-limitations are at hand.

Fish vaccines are available and confer some protection as the injection of mineral oil-adjuvanted bacterin vaccines induces a strong immune reaction reflected by high serum antibody levels^6,7^. However, furunculosis is still a serious threat to Danish maricultured rainbow trout (*Oncorhynchus mykiss*) and disease outbreaks occur even among vaccinated fish during summer periods with higher water temperatures^8^. Vaccination procedures may lead to marked production losses due to the starvation of fish before and after vaccination and the use of antibiotic treatments should be avoided due to environmental concerns. Therefore, alternative disease control approaches are required to ensure the sustainable expansion of rainbow trout aquaculture.

Selective breeding programs for disease resistant traits have a considerable potential^9^. For example, genetic resistance against infectious pancreatic necrosis virus (IPNV) was successfully improved in Atlantic salmon (*Salmo salar*) based on identification of a major QTL^10,11^. A major QTL in rainbow trout was found associated with resistance to *Vibrio anguillarum*^12^ and *Flavobacterium psychrophilum*^13^. QTL were linked to resistance against parasitic diseases such as amoebic gill disease (AGD) in Atlantic salmon^14^ and ciliate *Ichthyophthirius multifiliis* in rainbow trout^15^.

Attempts to identify brook trout (*Salvenius fontinalis*) strains resistant to *A. salmonicida* have been ongoing almost for a century^16–20^ showing a basis for selection programs based on significant genetic variation. This was also found for Atlantic salmon^21,22^ and the higher survival rates were later associated with MHC allele variation^23,24^. The protective mechanism was suggested to be elevated serum hemolytic activity in salmon^25^ and higher serum bactericidal activity in rainbow trout^26,27^.

GWAS has shown to be a promising tool to improve the genetic status of fish used for aquaculture purposes and the 57 K SNP panel^28^ has previously disclosed the genetic architecture of several traits of interest in rainbow trout^12,15,29–35^. Therefore the aim of this study was to assess the prospective of using GWAS to identify potential QTL for disease resistance against *A. salmonicida*. We genotyped 752 rainbow trout exposed to *A. salmonicida* through bath challenge during 13 days using the 57 K SNP panel and performed the GWAS for survival and time to death (TD) phenotypes to reveal QTL associated to *A. salmonicida* resistance. To gain insight of underlying mechanisms of resistance, we identified the positional candidate genes in the proximity of the QTL from the most recent assmbly of rainbow trout reference genome (USDA_OmykA_1.1 (GCF_013265735.2, NCBI)) and compared the expression profiles of immune-relevant genes in different fish groups.

## Results

### Mortality

**Figure 1:**
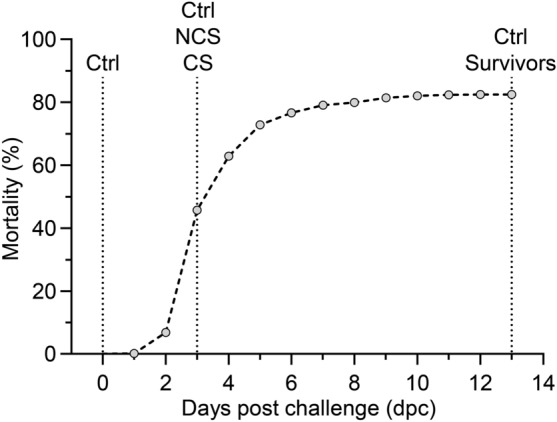
Kaplan–Meier plot (constructed with GraphPad Prism software) showing the development of mortality in 798 rainbow trout after waterborne infection with *Aeromonas salmonicida* subsp. *salmonicida* strain 111129–1/2. The curve represents cumulative mortality (%) of fish in 12 tanks. Dotted vertical lines mark the sampling time points for qPCR (Ctrl- non-exposed control fish, NCS—exposed fish with no clinical signs, CS—exposed fish with clinical signs, Survivors—exposed fish surviving the challenge).

The first moribund fish was recorded at 1 dpc (Fig. 1). From there on, the mortality increased sharply until 6 dpc (76%) and only few mortalities occurred thereafter. Mortality varied between the tanks to some extent (Supplementary Table S1) and the total mortality of 82% was recorded at 13 dpc.

### Genetic analysis

The results from REML analysis are presented in Table 1 and show that heritability to furunculosis infection was relatively high for survival (22% on the observed scale, 47% on the underlying scale of liability) and TD (36% on the observed scale). The estimated variance for additive genetic effects were 0.033 ± 0.010 and 4.975 ± 1.126 for survival and TD, respectively.

**Table 1:** Estimated variance of components from REML analysis of survival and time to death (TD) using a genomic animal model.

Factor	Survival	TD
Additive genetic variance	0.033 ± 0.010	4.975 ± 1.126
Residual variance	0.116 ± 0.008	9.031 ± 0.657
Phenotypic variance	0.149 ± 0.009	14.007 ± 0.967
Heritability (observed scale)	0.222 ± 0.059	0.355 ± 0.062
Heritability (underlying scale)	0.471 ± 0.125	–

**Figure 2:**
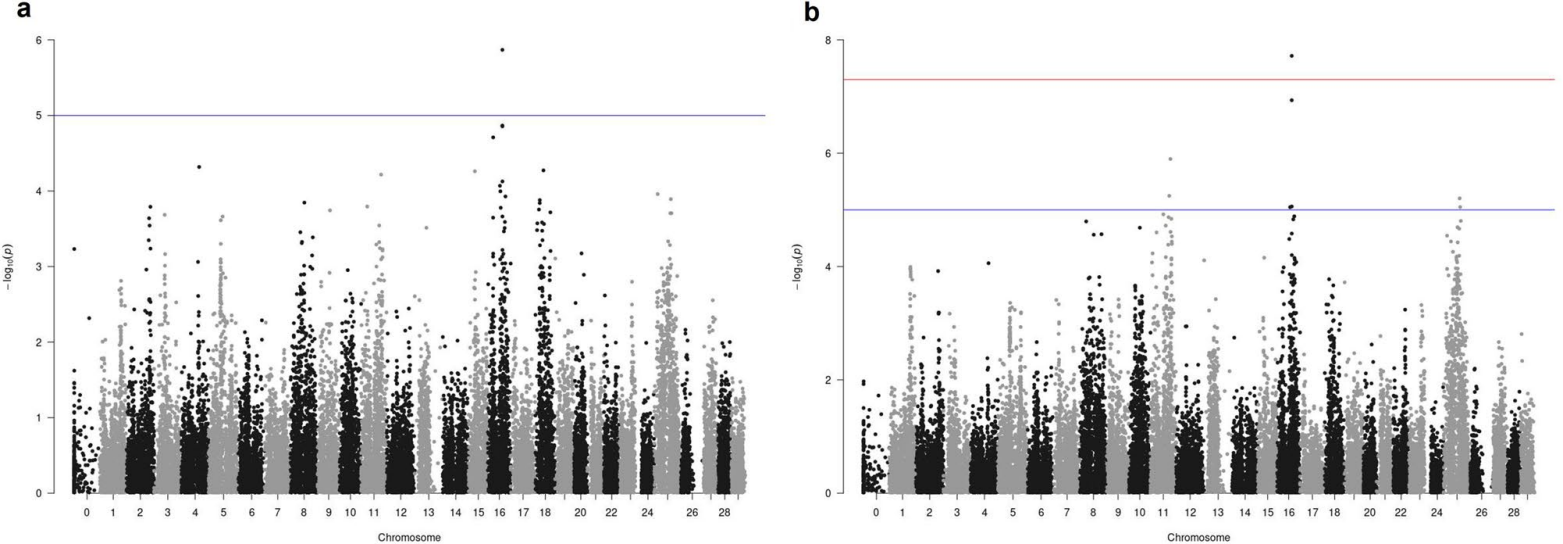
The Manhattan plot from LMM-LOCO GWAS of survival (**a**) and time to death (DT) (**b**) in rainbow trout after exposure to *A. salmonicida* subsp. *salmonicida.* The y-axis shows the p value for each SNP and x-axis individual SNPs. The red horizontal line represent the genome-wide significance threshold of p-value 5e−08 and the blue horizontal line indicates the suggestive level of significance with p-value of 1e−05.

The Manhattan plots for survival and TD (Fig. 2), as results of the LOCO-GWAS, indicate a significant QTL on chromosome 16, explaining 17% of the genetic variance for TD (Table 2). The same SNP (AX-89969631) came up as the most significant for both survival and TD, however, it was only clearly significant for TD (1.91e-8), likely due to a higher heritability for this trait.

**Table 2:** The top SNPs for TD and survival.

Chr	SNP	Position		Type	Freq	Effect	SE	P	Fr. genvar	Fr. phenvar
		USDA_OmykA_1.1 (GCF_013265735.2)	Omyk_1.0 (GCF_002163495.1)							
**Time to death (DT)**										
16	AX-89969631	48210249	44822522	C/T	0.84	− 1.76	0.31	1.91e−08	17%	6%
16	AX-89973999	48231736	44846688	G/T	0.80	− 1.49	0.28	1.16e−07	14%	5%
11	AX-89976064	65121697	61934614	C/A	0.25	− 1.34	0.28	1.26e−06	14%	5%
11	AX-89919380	61196699	58124529	C/T	0.15	− 1.42	0.31	5.66e−06	10%	4%
25	AX-89933102	10230926	49243042	C/T	0.55	− 1.04	0.23	6.25e−06	11%	4%
16	AX-89924175	48220966	44834124	G/A	0.68	− 1.16	0.26	8.72e−06	12%	4%
25	AX-89956080	11916756	50865587	C/A	0.41	1.06	0.24	8.92e−06	11%	4%
16	AX-89926977	42909875	39677850	G/A	0.09	1.69	0.38	8.96e−06	9%	3%
11	AX-89965839	42857280	39901842	G/T	0.22	− 1.42	0.32	1.20e−05	14%	5%
16	AX-89934677	56176558	52680845	C/T	0.52	1.30	0.30	1.30e−05	17%	6%
11	AX-89942657	59019488	56061980	C/A	0.84	1.33	0.31	1.35e−05	10%	3%
11	AX-89963218	67994309	64707318	C/A	0.88	1.47	0.34	1.44e−05	9%	3%
16	AX-89919325	53039122	49570500	C/T	0.43	− 1.10	0.25	1.47e−05	12%	4%
25	AX-89957036	13953432	52861958	C/T	0.50	− 0.88	0.20	1.57e−05	8%	3%
**Survival**										
16	AX-89969631	48210249	44822522	C/T	0.84	− 0.16	0.033	1.35e−06	20%	5%
16	AX-89924175	48220966	44834124	G/A	0.68	− 0.12	0.03	1.36e−05	19%	4%
16	AX-89973999	48231736	44846688	G/T	0.80	− 0.13	0.03	1.39e−05	16%	4%
16	AX-89976723	19107648	16278865	C/A	0.55	− 0.10	0.02	1.95e−05	14%	3%
4	AX-89965612	12013837	54733880	G/A	0.14	0.16	0.04	4.81e−05	19%	4%
15	AX-89918838	22601764	19112526	G/A	0.44	0.11	0.03	5.48e−05	18%	4%
11	AX-89976064	65121697	61934614	C/A	0.25	− 0.12	0.03	6.07e−05	16%	3%
16	AX-89964054	48349717	44952397	C/A	0.47	− 0.11	0.03	7.48e−05	18%	4%
16	AX-89955003	40411668	37206251	C/A	0.90	− 0.15	0.04	8.53e−05	14%	3%

The two last columns give the estimated fraction of genetic and phenotypic variance explained by each SNP. SNPs are listed based on significance.

**Figure 3:**
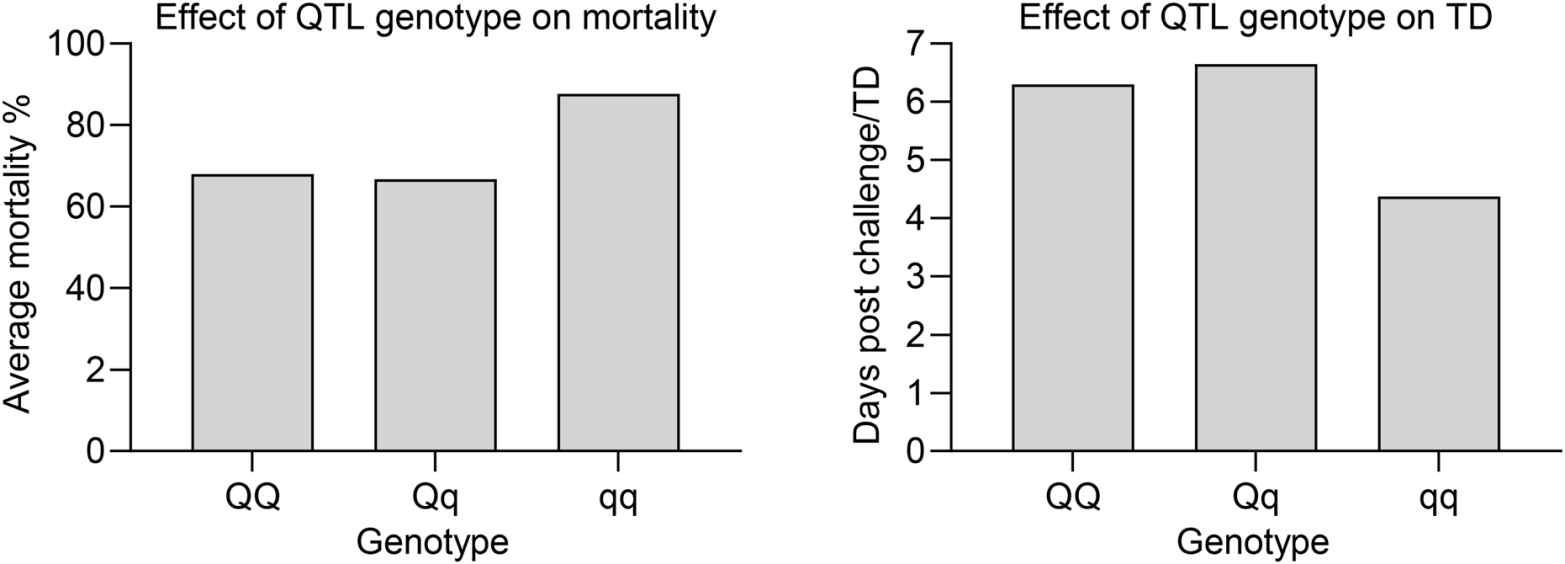
Average observed mortality for different genotypes: (**a**) survival and (**b**) time to death (TD) after challenge with *A. salmonicida* subsp. *salmonicida* for the top SNP (AX-89969631) at chromosome 16.

The calculation of the average observed mortality for fish having different genotypes for the most significant SNP is presented in Fig. 3. The favorable homozygote (QQ) and heterozygote (Qq) had an overall mortality of 66% while the mortality for the unfavorable homozygote (qq) was 88%. A similar tendency was observed for TD.

### Candidate genes

Positional candidate genes within the 1000 Kb window (47 750 K–48 750 K) of the most significant SNPs (AX-89969631 and AX-89973999) associated with resistance on chromosome 16 (NC_048580.1) was identified using the latest assembly of rainbow trout genome USDA_OmykA_1.1 (GCF_013265735.2) in NCBI-Genbank. Among the 33 genes located in this region, we found 30 protein-coding genes, 1 pseudogene and 2 non-coding genes (Table 3). The most significant SNP (AX-89969631) was located in the LOC118939552 region of non-coding RNA while the gene encoding PRA1 family protein 3 mapped into the second most significant SNP (AX-89973999).

**Table 3:** Positional candidate genes within the 1000 Kb window (47,750 K–48,750 K) of the two most significant SNPs (AX-89969631 and AX-89973999) from the latest assembly of the rainbow trout genome USDA_OmykA_1.1 (GCF_013265735.2) in NCBI-Genbank.

Gene ID	Description	Type	Location	Strand
LOC110492148	Calmodulin-binding transcription activator 1	Protein coding	47409235–47908274	−
LOC110492150	dnaJ homolog subfamily C member 11	Protein coding	47918157–47927478	−
thap3	THAP domain containing, apoptosis associated protein 3	Protein coding	47918157–47927478	−
LOC110492151	F-box only protein 6	Protein coding	47932110–47950665	+
LOC110492153	Cell division control protein 42 homolog	PROTEIN coding	47953418–47956955	−
LOC118939548	Uncharacterized	ncRNA	47981859–47988731	+
LOC110492154	Lamina-associated polypeptide 2, isoforms beta/delta/epsilon/gamma	Protein coding	48006228–48010038	+
LOC118939547	N-fatty-acyl-amino acid synthase/hydrolase PM20D1.2-like	Protein coding	48010169–48026769	+
LOC118939549	Acidic mammalian chitinase-like	Protein coding	48031902–48036060	−
LOC118939550	Neurofascin-like	Pseudo	48042033–48074973	−
LOC118939551	Calcium-independent phospholipase A2-gamma-like	Protein coding	48076504–48087589	−
LOC110492156	Uncharacterized protein At5g50100, chloroplastic	Protein coding	48089427–48093940	−
LOC110491082	Monocarboxylate transporter 2-like	Protein coding	48101686–48113512	+
LOC110492157	Chemokine-like protein TAFA-1	Protein coding	48145652–48171888	+
LOC110491083	chemokine-like protein TAFA-4	Protein coding	48184977–48196697	−
**LOC118939552**	**Uncharacterized LOC118939552**	**ncRNA**	**48208293–48212220**	**−**
LOC110492159	NEDD8-activating enzyme E1 catalytic subunit	Protein coding	48220758–48230211	−
**LOC110492162**	**PRA1 family protein 3**	**Protein coding**	**48231344–48235518**	** + **
LOC110492161	Leiomodin-3	Protein coding	48234990–48241496	−
LOC110492160	FERM domain-containing protein 4B	Protein coding	48242058–48272289	−
LOC110492164	Microphthalmia-associated transcription factor	Protein coding	48282132–48331926	+
LOC110492165	2-epi-5-epi-valiolone synthase	Protein coding	48333920–48343017	−
LOC110492167	myoD family inhibitor domain-containing protein 2-like	Protein coding	48343132–48347793	−
LOC110492168	forkhead box protein P1-B	Protein coding	48348467–48371945	−
LOC110492170	Eukaryotic translation initiation factor 4E type 3	Protein coding	48372037–48383460	−
LOC110492169	Serine/threonine-protein phosphatase 4 regulatory subunit 2-A	Protein coding	48383542–48393271	+
LOC110491084	Guanylyl cyclase-activating protein 2	Protein coding	48400096–48402355	+
oard1	O-acyl-ADP-ribose deacylase 1	Protein coding	48403279–48405646	+
LOC110492174	Synaptotagmin-2	Protein coding	48407064–48483333	+
LOC110492173	Protein phosphatase 1 regulatory subunit 12B	Protein coding	48483470–48521507	−
LOC110492176	Neurofilament medium polypeptide	Protein coding	48525042–48533176	−
LOC110491085	Immunoglobulin-like and fibronectin Type III domain-containing protein 1	Protein coding	48547180–48548167	−
LOC110492177	Metabotropic glutamate receptor 4	Protein coding	48650173–48970488	−

The genes highlighted in bold include the two most significant SNPs.

### Immune gene expression

**Figure 4:**
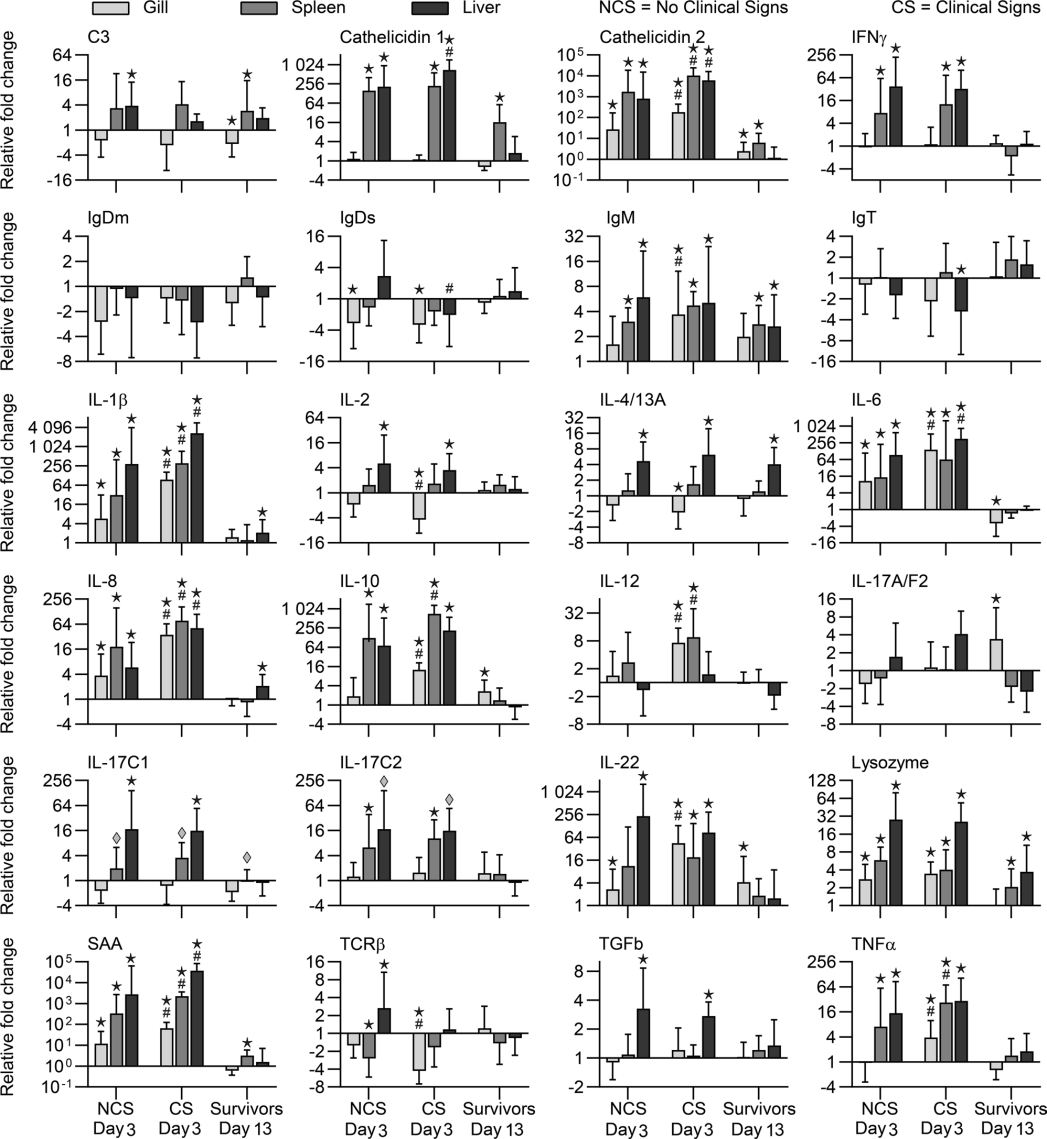
Different expression patterns of cytokine and effector molecule genes after infection with *A. salmonicida* subsp. *salmonicida* in rainbow trout. NCS: exposed fish with no clinical signs, CS: exposed fish with clinical signs, Survivors—exposed fish surviving the challenge at 13 dpc. Star: Significant differences (p < 0.05) between non-exposed (control) groups and exposed groups—NCS, CS and survivors. ^#^Significant differences (p < 0.05) between the NCS and CS groups at 3 dpc. Diamond: Significant differences (p < 0.05) with non-parametric Mann–Whitney test were applied for fish groups that had less than 3 positive Cq values.

Significantly expressed immune-relevant genes are displayed in Fig. 4 reflecting different involvement of three different organs in exposed fish when compared to non-exposed controls. The majority of genes were highly up-regulated in the liver, while less regulated genes were recorded in the gills. The majority of the significant regulations occurred at 3 dpc in the fish exposed to bacteria and the highest regulations were found in the fish showing clinical signs (CS). The *A. salmonicida* exposed fish that were not showing clinical signs (NCS) exhibited similar regulation of the same genes, although to a lower degree.

#### Expression at 3 dpc

All the genes except those encoding IgT, IgDs and IgDm were significantly regulated in at least one of the sampled organs of infected fish at 3 dpc.

##### Liver

Genes encoding innate immune effector molecules (C3, cathelicidins, lysozyme, SAA) were significantly up-regulated in the liver of infected fish. Up-regulation of the gene encoding IgM was recorded whereas other immunoglobulin genes did not show a clear involvement. A substantial significant up-regulation occurred for the genes for the main pro-inflammatory cytokines (IL-1β, IL-6, IL-8, TNFα, IFNγ), regulatory cytokines (IL-10, TGFβ) and cytokines involved in functions of different Th cell subsets (IL-2, IL-3/14a, IL-17C1, IL-17C2, IL-22, TCRβ).

##### Spleen

The majority of genes in the spleen were generally expressed at a lower level when compared to the liver, but the genes encoding cathelicidin 2, IL-8, IL-10 and IL-12 showed a higher-fold splenic up-regulation. Genes encoding IL-4/13a, IL-17A7/F2, IL-22, TGFβ and C3 showed no significant regulation in the spleen.

##### Gills

The gene expression in the gills was primarily seen in fish with clinical signs. In some cases, we found that genes significantly up-regulated in one or both of the other organs, were significantly down-regulated in gills (IL-2, IL-4/13a, C3) or showed no significant regulation (IL-17A/F2, IL-17C1, IL-17C2, TGFβ, IFNγ, cathelicidin 1). Cytokine genes (IL-1β, IL-6, IL-8, TNFα, IL-10, IL-12, IL-22) and innate immune genes encoding for cathelicidin 2, lysozyme and SAA were in most cases significantly up-regulated in infected groups. Genes encoding immunoglobulins were mainly down-regulated, with the exception of a significant up-regulation for IgM in the gills of fish showing clinical signs.

#### Expression at 13 dpc

In the late phase of infection, marked by a flattened mortality curve, surviving fish showed a lower gene expression level.

##### Liver

Genes encoding IL-1β, IL-4/13a, IL-8, lysozyme and IgM were moderately up-regulated in surviving fish.

##### Spleen

Genes encoding C3, cathelicidins, lysozyme, SAA, IgM and IL-17C1 were significantly up-regulated at this late infection time-point.

##### Gills

The gene encoding C3 was slightly but significantly down-regulated, and the gene for cathelicidin 2 was up-regulated in fish surviving the infection. A slight significant up-regulation occurred also for the cytokine genes encoding IL-10, IL-17A/F2 and IL-22, while the gene for IL-6 was down-regulated.

### Bacterial load

**Figure 5:**
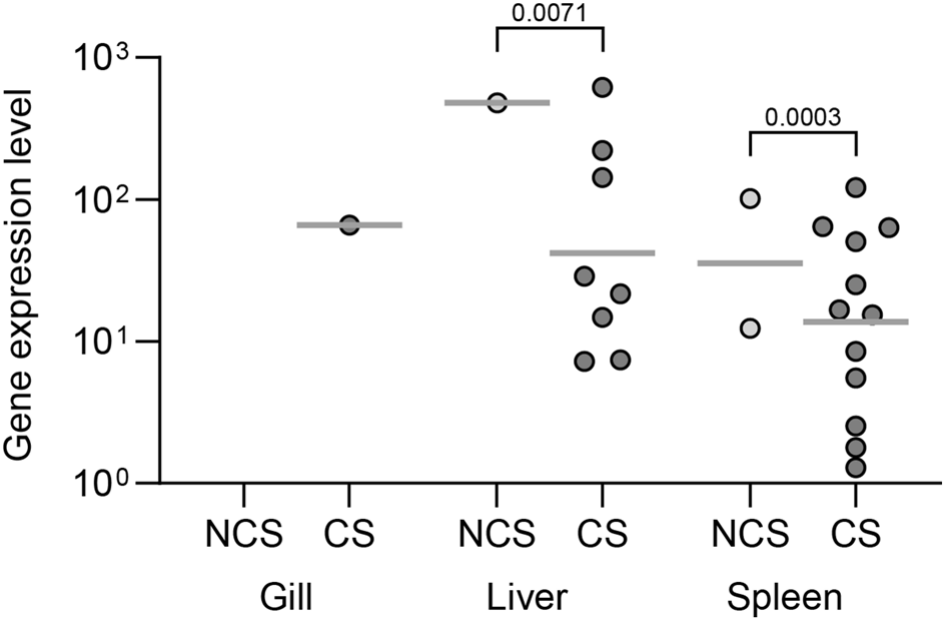
Level of *A. salmonicida* subsp. *salmonicida* gene (*aopO*) transcripts in different organs of fish at 3 days post challenge (dpc): NCS: exposed fish with no clinical signs, CS: exposed fish with clinical signs. Fifteen fish from each group were investigated. Number above the square brackets indicate p values between groups. For the NCS group, only few fish showed positive Cq values, therefore qualitative assessment was applied (presence or absence of Cq values) and data was analyzed with nonparametric Mann–Whitney test.

Bacterial infection levels at 3 dpc are illustrated in Fig. 5. Fish with clinical signs had higher level of bacterial transcripts compared to the fish that were exposed to bacteria but had no clinical signs. In general, only few fish without clinical signs had bacterial transcripts in their organs (1/15 and 2/15 for liver and spleen, respectively). No bacterial transcripts were found in the organs of surviving fish at 13 dpc.

## Discussion

Furunculosis is among the main bacterial diseases affecting rainbow trout in Danish marine aquaculture farms. Vaccination has shown to protect salmonids under certain conditions^6^ but disease outbreaks among vaccinated fish often occur in Danish rainbow trout mariculture farms when water temperatures exceed 20 °C^8^. This calls for novel approaches of disease prevention and in this context, genetic breeding of disease resistant rainbow trout may be a possible way forward. In the present study we searched for a QTL associated with furunculosis resistance in rainbow trout applicable for new breeding programs. In addition, we aimed to detect the genetic regions and genes that explain the resistance and supported this approach with immune gene expression studies of susceptible and resistant trout. The genome-wide association study involving 752 rainbow trout exposed to *A. salmonicida* showed that heritability of furunculosis resistance in trout was relatively high, both regarding survival and time to death phenotypes. Mortality after the bath challenge was high (82%), but declined and stabilized towards the end of the challenge period. This indicated that the survivors are not just fish with longer incubation period, but truly resistant individuals. This notion should, however, be further elucidated by increasing samplings during the entire study period (and confirm absence of bacteria in survivors) and by re-exposing survivors to *A. salmonicida* to confirm true resistance. A significant QTL was found on chromosome 16 carrying SNPs being significant for both survival and TD. However the most significant SNP reached the genome-wide significance threshold only for TD, likely due to the higher heritability of the trait. This SNP explained a moderate fraction (17%) of genetic variance for TD suggesting that the majority of the genetic variation is probably explained by oligogenic effects. In such cases genomic selection may be more efficient than marker-assisted selection for this QTL only.

The favorable homozygote and the heterozygote had lower mortality compared to the unfavorable homozygote for both survival and TD, possibly indicating dominance of the favorable allele. However this is uncertain as the frequency of favorable allele was low (16%). Thus, there is a large room for improvement of natural resistance in rainbow trout populations. Based on the present work, relatively good protection could be achieved by avoiding unfavorable homozygotes in commercial production (for example by restricting sires to be homozygous for the favorable allele).

To learn more about the underlying mechanisms of resistance, we identified the positional candidate genes within a 1000 Kb window of the two most significant SNPs on chromosome 16. The most significant SNP (AX-89969631) that explained the greatest proportion of genetic variance was located in the second intron of an uncharacterized non-coding RNA gene (LOC118939552). The second most significant SNP (AX-89973999) was located in the first intron of the gene encoding PRA1 family protein 3 (Gene ID 110492162). This gene has previously been suggested to be linked to *A. salmonicida* resistance in turbot^36^ and a its paralogue gene was listed among positional candidate genes associated to *Flavobacterium columnare* resistance in rainbow trout^33^. The PRA1 family protein 3 in humans reduces viral anti-apoptotic activity and thus has an important role in host-mediated apoptosis and immune response^37^. The region close to SNP AX-89973999 contains another gene identified in the QTL region with the largest effect on resistance to *F. columnare*^33^. This gene encodes for a FERM domain-containing protein 4B known as a scaffolding protein regulating epithelial cell polarization^38^. The gene located between the two most significant SNPs encodes for the NEDD8-activating enzyme E1 catalytic subunit. Neddylation is a post-translational protein modification that effects gene regulation, cell survival, organ development, stress- and immune response^39^. The blocking of neddylation by the NEDD8 activating enzyme E1 inhibitor has shown to suppress antiviral response in zebrafish and increased the sensitivity to spring viremia of carp virus (SVCV) infection^40^. In close proximity to the SNP location, we found genes encoding chemokine-like protein TAFA-1 and TAFA-4, both belonging to a family of small secreted proteins. These proteins, distantly related to CC chemokines, are primarily expressed in the central nervous system (CNS) and their biological functions might involve functioning as brain-specific chemokines regulating immune and nervous cells^41^.

Other genes adjacent to the two most significant SNPs comprise the gene encoding leiomodin-3 and microphthalmia-associated transcription factor (MITF). The leiomodin-3 protein has a role in organization of actin filaments in skeletal muscle in zebrafish^42^ and MITF regulates the expression of several other genes and thereby the lineage-specific regulation of several cell types including melanocytes, mast cells, and osteoclasts^43^. The latter gene also plays a role for cell homeostasis, apoptosis and cell cycle^44^.

More distantly located genes that have been reported to be involved in immune response in different fish species include the forkhead box protein P1-B (FOX B-1)—a member of a large family of transcription factors well-characterized in mammals. FOX B-1 has a pivotal role in human and mouse B-cell development and is involved in the expression of recombination-activating genes (RAG) in pro-B cells and in V(D)J recombination^45^. In zebrafish, FOX B-1 has shown to be an important transcription factor in the development of CNS and other organs^46^. In rainbow trout, FOX P1 was found up-regulated after *A. salmonicida* challenge in the gills, contributing in the regulation of brachial immunity in this species^47^.

It is likely that several genes have a role in the resistance to furunculosis, some related to immune defense and others engaged in different stages of pathogen-host interaction. It was recently suggested that the epithelial cadherin (cdh1) gene, encoding a protein that bind IPNV virions during virus internalization, is a major determinant of the resistance to IPNV in Atlantic salmon^48^. This frames that the identification of genes underlying the QTL in disease resistance requires knowledge of the intricate and complex interactions between the host and the pathogen^49^.

None of the positional candidate genes identified in the present study were targeted in the qPCR analysis. However, the gene expression profile gave an overview of the innate and adaptive immune genes generally involved in bacterial infection. It cannot be excluded that one or more of the positional candidate genes mentioned above play a role in the antibacterial response. We examined the immune gene expression response in three different organs: the gills, as the primary site of bacterial entry; the liver, functioning as the main producer of complement- and acute phase proteins; and lastly the spleen, responsible for antigen presentation and immune regulation^50^.

The highest transcriptional changes post-exposure took place in the liver and spleen. The gene regulation response in various organs could be related to the level of bacterial transcripts in these organs at 3 dpc. In the gills, where the gene regulation was lower compared to other organs, only a single fish was detected with bacterial gene transcripts. Both liver and spleen, showing a strong gene-expression response, were generally positive for bacterial gene transcripts. It was also evident that fish exhibiting clinical signs reacted much stronger when compared to fish without any visible disease signs. Thus, the magnitude of invasion reflects the strength of the response. The surviving fish had no bacterial transcripts in the gills, liver and spleen, suggesting that these fish are able to clear the infection in these organs. However, further studies should investigate if the survivors were free from infection during the entire challenge period. Re-challenge of survivors would also confirm the pathogen-free status. *A. salmonicida* has been found in the gut and brain of asymptomatic carrier fish^51^ suggesting that a low number of pathogens are kept at distance by the innate (SAA, C3, cathelicidins, lysozyme) and adaptive (IgM) immune genes activated in the resistant surviving fish.

The early infection (3 dpc) triggered a strong transcriptional up-regulation of many immune genes in different organs including those encoding pro-inflammatory cytokines IL-1β, IL-6 and TNFα and other cytokines involved in the development of adaptive immunity (IL-8, IL-10, IL-22, IL-17C2, INFγ). The clear up-regulation of previously mentioned immune markers is consistent with earlier studies in trout exposed to *A. salmonicida*^47,52,53^ and other bacterial pathogens^12,35^. Along with the inflammation induced we saw a marked up-regulation of SAA, C3 and several other components of the innate immune system (lysozyme and cathelicidins). IgM was the only immunoglobulin found to be up-regulated from the early state of infection. This confirms previous suggestions that antibodies are key players in protection against *A. salmonicida*^7^. We clearly observed a systemic immune response to *A. salmonicida* infection in trout but it is noteworthy that susceptible fish achieving a high pathogen load and with external clinical signs generally responded stronger than fish resisting the challenge. However, the surviving fish could have resisted the infection based on one or several factors including possible candidate genes associated with specific SNPs, innate immune components and IgM.

## Conclusion

This is to our knowledge the first study that aims to identify the genomic regions associated with *Aeromonas salmonicida* resistance in rainbow trout by using the 57 K SNP array. We found that resistance to *A. salmonicida* is moderately heritable and showed an oligogenic architecture. We discovered a significant QTL on cromosome 16 and the most significant SNP explained 17% of the genetic variance in resistance to furunculosis. Rainbow trout infected with *A. salmonicida* exhibited a notable up-regulation of cytokine and innate immune factor genes 3 days after bacterial challenge in gills, liver and spleen, indicating an induction of systematic immune response to the infection. Future GWAS analysis should further validate the oligogenic nature of the resistance to *A. salmonicida*. In addition, comparative transcriptome analyses could provide more detailed information on gene expression differences between susceptible versus resistant fish and the possible connection with detected candidate genes. Additional gene editing techniques may be applied to validate the functional role of different genes in resistance in trout and other host species.

## Materials and methods

### Ethics statement

Infection procedures were performed under the license no. 2019-15-0201-01614 issued by the Experimental Animal Inspectorate, Committee for Experimental Animals, Ministry of Environment and Food, Denmark. ARRIVE guidelines and ethical guidelines of the University of Copenhagen were followed securing that fish were monitored every second hour around-the-clock. Fish showing clinical signs (loss of equilibrium, irregular swimming, skin hemorrhages, severe discoloration) were immediately euthanized with overdose (300 mg/l) of tricaine methane sulphonate (MS222, Sigma-Aldrich, Søborg, Denmark) and recorded as mortalities.

### Fish

An outbred population of 60 half-sibling families (12–13 fish from each family) of rainbow trout was used for the experiments (Hallesø trout farm, Aquasearch ova ApS, Jutland, Denmark). Disinfected eyed rainbow trout eggs, were transported to Aqua Baltic pathogen-free hatchery^54^ (Nexø, Denmark) and hatched at 7 °C during 14 days. Fish were reared in 700 l tanks with recirculating municipal water (12 °C) and fed 1% biomass of dry pelleted feed (INICIO 917 BioMar A/S, Brande, Denmark) daily. The juvenile fish (1800 degree-days post-hatch, average body weight 8 g and length 8.5 cm) were transported to the experimental fish facility at the University of Copenhagen (Frederiksberg, Denmark). The fish for the challenge study were allocated in twelve 150 l tanks equipped with internal biofilters (20 l/min EHEIM, Deizisau, Germany), accommodating approximately 70 fish per tank. Fish were acclimatized at 19 °C for 14 days prior the challenge in order to conduct the infection study at temperature similar to outbreak conditions. Fish were fed 1% biomass daily (INICIO 917, BioMar) and the rearing conditions were kept constant at pH 7.6, nitrite < 0.01 mg/l, nitrate < 50 mg/l (Tetra GmbH, Melle, Germany), ammonia < 0.5 mg/l (Hach, Loveland, CO, USA) and 30% water was replenished every day. Non-exposed control fish were kept under corresponding conditions in a separate room during the study.

### Challenge

Head kidney swabs from 5 freshly euthanized fish were plated onto 5% blood agar plates (SSI Diagnostica, Hillerød, Denmark) before challenge to confirm the fish were free from bacterial infection^55^. Fish (n = 798) were exposed to *A. salmonicida* subsp. *salmonicida* strain 111129-1/2 by bath challenge. The 48 h bacterial culture (total volume 5 l, 4.7 × 10^8^ cfu/ml) was added (400 ml to each fish tank carrying 30 l aerated water) whereby the fish were exposed to a bacterial concentration of 6.26 × 10^6^ cfu/ml. Fish were kept exposed for 6 h whereafter tanks were filled with tap water to reach the volume of 150 l. Fish were then observed for occurrence of any disease signs for 13 days.

### Sampling

#### Mortality recordings

Mortality was recorded from the exposure time point to 13 days post challenge (dpc). The remaining fish were sampled at 13 dpc and recorded as survivors.

#### Sampling for genotyping

Samples for genotyping were taken from a total of 798 fish throughout the course of infection. Fish showing clinical signs (653) were sampled as susceptible and at 13 dpc all fish surviving the challenge (145) were sampled as resistant. For DNA-typing, a circular tissue piece (Ø 2.75 mm) was taken from the tail fin of each fish using punching scissors (AgnTho’s AB, Lidingö, Sweden) and transferred to lysis buffer (Vaxxinova Norge, Bergen, Norway) for subsequent DNA purification and genotyping according to Karami et al.^12^.

#### Genotyping

The high-density 57 K single nucleotide polymorphism (SNP) chip array developed for rainbow trout^28^ by ®Affymetrix, San Diego, CA, USA, was used for genotyping. All analysis was conducted according to the Axiom platform Assay-Automated-Workflow-User-Guide^56^.

#### Sampling for gene expression analysis

Gills, liver and spleen were sampled from 15 non-exposed control fish at day zero. At 3 dpc samples were obtained from 15 non-exposed control fish, 15 exposed fish showing clinical signs (CS) and 15 exposed fish showing no clinical signs (NCS). At 13 dpc when no fish showed clinical signs of disease, 15 non-exposed control fish and 15 exposed but surviving fish were sampled. Samples were fixed in RNAlater (R0901, Sigma-Aldrich), placed at 4 °C for 24 h and subsequently stored at -20 °C until further processing.

### Quantitative RT-PCR (qPCR)

The genes investigated by qPCR analysis were encoding immune-relevant molecules and included SAA, C3, lysozyme, cathelicidins, IFNγ, IgM, IgT, IgD, IL-1β, IL-2, IL-4/13a, IL-8, IL-10, IL-12, IL-17A/F2, IL-17C1, IL-17C2, IL-22, TCRβ, TGFβ, TNFα. Primers and Taq-Man probes applied are listed in Supplementary Table S2 and the gene expression analysis was performed as previously described^35^. In brief, samples from gills, liver and spleen were homogenized (Tissue-lyser II, Qiagen, Vedbæk, Denmark) and RNA was extracted by GenEluteTM mammalian RNA kit (RTN350, Sigma-Aldrich). The cDNA was synthesized in T100 thermocycler (Biorad, Copenhagen, Denmark) using Oligo d(T)16 primers and TaqMan® reverse transcription reagents (N8080234, Thermo Fischer Scientific, Roskilde, Denmark). Quantitative PCR assays were performed using AriaMx Real-Time PCR machine (G8830A-04R-010, AH diagnostics AS, Denmark). Primers and Taq-Man probes targeting immune-relevant rainbow trout genes were synthesized at TAG Copenhagen AS, Copenhagen, Denmark. The 12.5 µl total volume reactions consisted of 2.5 μl cDNA, 6.25 μl Brilliant III Ultra-Fast QPCR Master Mix (600,881, AH Diagnostics AS, Tilst, Denmark), 1.0 μl primer–probe mixture (10 μM forward primer and reverse primer, 5 μM Taq-Man probe) and 2.75 μl RNase-free water (Invitrogen, Denmark). Reverse transcriptase minus and negative controls were used for every plate setup. The *A. salmonicida* infection level was monitored by quantifying the bacterial load in different organs using qPCR primers and probes targeting the *aopO* gene (DQ386862) of *A. salmonicida*^57^. The bacterial transcript level was estimated as 10^7^ × 2^−∆Cq^ based on bacterial cDNA as previously described for *Yersinia ruckeri*^35^. It should be noted that dead or inactive bacteria is not detected by this method.

### Data analysis

#### Survival

GraphPad Prism version 9.0.0. (GraphPad Software www.graphpad.com) was used to estimate the cumulative mortality rates by Kaplan-Mayer survival analysis.

#### Gene expression

All qPCR assays exhibited efficiencies within 100% ± 5% and the simplified 2^-ΔΔCq^ method was used for data analysis^58^. The average of three genes (*arp*, *elf1α* and *β-actin*) was chosen for normalization and as internal calibrator, using NormFinder^59^. For gene expression analysis at 3 dpc, 2 challenged groups and one control group were compared using one-way ANOVA with Tukey’s test and for analysis at 13 dpc, survivors and control fish were compared with student’s t-test. Minimum twofold regulations were considered substantial and differences between groups were tested with 2-tailed t-test (p < 0.05). Qualitative assessment was applied for the groups that had less than 3 positive Cq values. For that purpose, presence or absence of Cq values was analyzed with nonparametric Mann–Whitney test (p < 0.05).

#### Genetic analysis

Only fish of high genotype quality were included in the genetic analysis. Therefore, 46 fish were discarded from the study before genetic analysis, resulting in a dataset of 752 fish (out of 798 fish)—138 survivors and 614 dead individuals. The resistance traits investigated were survival and time to death (TD—survival interval between infection and death). SNPs included in the study were restricted to 32 205 loci and showed all three possible genotype clusters (PolyHighRes) by Thermo Fisher Array Power Tools software. Statistical analysis was performed according to our latest QTL study^12^. Shortly, the genomic relationship matrix (GRM) was computed using the Genome-Wide Complex Trait Analysis (GCTA) software^60^. The data set was initially analyzed with a simple genomic animal model using restricted maximum likelihood (REML): $y=Xb+g+e$

Where **y** is a vector of phenotypes (0/1 for survival or TD), b is the vector of fixed effects (12 different tanks) with associated incidence matrix X, $g\sim N(0,G{\sigma }_{g}^{2})$ is a vector of additive genetic (polygenic) effects, $G$ is the genomic relationship matrix, computed from all high-quality markers, ${\sigma }_{g}^{2}$ is the polygenic variance, $\mathrm{e}\sim N(0,I{\sigma }_{e}^{2})$ is a vector of random residual effects and ${\sigma }_{e}^{2}$ is the residual variance. The genetic variance was calculated (assuming that the estimated SNP effect is the actual effect) using a following formula: $V\left(SNP\right)=2\times freq\left(1-freq\right){b}^{2}$, where freq is the allele frequency and b is the estimated SNP effect.

Secondly, a leave-one-chromosome-out genome-wide association study (LOCO-GWAS) was performed using the same model as above but extended with individual SNP effects. Here, polygenic effects on all other chromosomes, except the one being currently tested, was accounted for. The rationale using the LOCO-GWAS model is that the model takes into account the potential stratification of the tested population, which is especially relevant to consider in farmed fish with strong relationship structures. In the current model, an animal genetic (polygenic) effect was therefore included, with animal effects having a covariance structure throughout the GRM along with fixed effects on the SNP that is currently tested (all SNPs are tested as separate effects, one-by-one). Thus, the SNP effects were solutions from the linear mixed LOCO-GWAS model which included a fixed overall mean, a fixed regression using the SNP being tested as a covariate (the estimated SNP effect is the solution for this regression coefficient) and a random polygenic animal effect (with GRM estimated from all chromosomes except the one being tested).

Using a linear model, heritability was estimated on the observed scale. The heritability estimates for survival were transformed to the underlying liability scale using a build-in option of GCTA software based on the theory and formulas presented in Lee et al.^61^.

For the SNPs to be significantly associated with resistance, Bonferroni correction was applied (i.e. $0.005/N$, where N refers to the number of SNPs).

## Supplementary Information

Supplementary Information.

## Data Availability

The datasets generated and data analysed during the current study are made available from the corresponding author on request.
