# Supplementary material for: Whole-genome association study searching for QTL for Aeromonas salmonicida resistance in rainbow trout
Source: Sci Rep. 2021 Sep 8;11:17857. doi: 10.1038/s41598-021-97437-7 (PMC8426485; doi:10.1038/s41598-021-97437-7)
Supplement: Supplementary file 1 — Supplementary Information. [file 41598_2021_97437_MOESM1_ESM.docx]

**Whole-genome association study searching for QTL for *Aeromonas salmonicida* resistance in rainbow trout**

***Moonika H. Marana^1*^, Asma M. Karami^1^, Jørgen Ødegård^2^, Shaozhi Zuo^1^, Rzgar M. Jaafar^1^, Heidi Mathiessen^1^, Louise von Gersdorff Jørgensen^1^, Per W. Kania^1^, Inger Dalsgaard^3^, Torben Nielsen^4^ and Kurt Buchmann^1^***

*^1^Laboratory of Aquatic Pathobiology, Department of Veterinary and Animal Sciences, Faculty of Health and Medical Sciences, University of Copenhagen, Frederiksberg C., Denmark*

*^2^Aquagen, Norway*

*^3^Institute of Aquatic Resources, Technical University of Denmark, Kgs. Lyngby, Denmark*

*^4^Aquasearch ova ApS, Jelling, Denmark*

**Supplementary Table S1**

Mortality in rainbow trout after waterborne infection with *Aeromonas salmonicida* subsp. *salmonicida* strain 111129-1/2 in 12 replicate tanks.

| **Tank** | **Mortality %** | **2** | **3** | **4** | **5** | **6** | **7** | **8** | **9** | **10** | **11** | **12** |
| --- | --- | --- | --- | --- | --- | --- | --- | --- | --- | --- | --- | --- |
| **1** | **84.3** | ns | ns | ns | ns | ns | ns | <0.001 | <0.0001 | <0.05 | ns | ns |
| **2** | **83.6** |  | ns | ns | ns | ns | ns | <0.001 | <0.0001 | <0.05 | ns | ns |
| **3** | **85.3** |  |  | ns | ns | ns | ns | <0.01 | <0.0001 | ns | ns | ns |
| **4** | **90** |  |  |  | ns | ns | ns | <0.0001 | <0.0001 | <0.001 | ns | ns |
| **5** | **87.1** |  |  |  |  | ns | ns | <0.0001 | <0.0001 | <0.001 | ns | ns |
| **6** | **88.6** |  |  |  |  |  | ns | <0.0001 | <0.0001 | <0.01 | ns | ns |
| **7** | **88** |  |  |  |  |  |  | <0.001 | <0.0001 | <0.01 | ns | ns |
| **8** | **73.9** |  |  |  |  |  |  |  | <0.05 | ns | <0.0001 | ns |
| **9** | **56.2** |  |  |  |  |  |  |  |  | <0.01 | <0.01 | <0.001 |
| **10** | **74.3** |  |  |  |  |  |  |  |  |  | <0.01 | ns |
| **11** | **89.4** |  |  |  |  |  |  |  |  |  |  | ns |
| **12** | **86.7** |  |  |  |  |  |  |  |  |  |  |  |

**Supplementary Table S2**

Primers and probes used for qPCR assays. All nucleotides are from 5’ end (labeled with FAM) to 3’ end (labeled with BHQ1). The qPCR assays were optimized to have annealing temperature of 60°C and 100% ± 5% efficiencies.

| **Gene**  **GenBank acc.no.** | **Length**  **Bp** | **Primers**  **5’end to 3’end** | **Probes**  **5’end to 3’end** | **References** | **Genomic localisation** | |
| --- | --- | --- | --- | --- | --- | --- |
|  |  |  |  |  | **Localisation** | **GenBank acc.no.** |
| ^R^ *arp*  AY505012 | 106 | Fwd: GAAAATCATCCAATTGCTGGATG  Rev: CTTCCCACGCAAGGACAGA | CTATCCCAAATGTTTCATTGTCGGCGC | [1] | chr17:40340265 to 40340360 | NC_035093 |
| ^R^ *β-actin*  AB196465 | 241 | Fwd: ACATCAAGGAGAAGCTGTGCTAC  Rev: TACGGATGTCCACGTCACAC | CCTCTCTGGAGAAGAGCTACGAGCTG | [2]  Probe [3] | chr:1740340265 to 40340360 | NC_035093 |
| ^R^ *elf-1α*  [AF498320](http://www.ncbi.nlm.nih.gov/entrez/viewer.fcgi?db=nucleotide&val=20269865) | 63 | Fwd: ACCCTCCTCTTGGTCGTTTC  Rev: TGATGACACCAACAGCAACA | GCTGTGCGTGACATGAGGCA | [4] | chr14:30894810 to 30894861 | NC_035090 |
| *c3.3* & *c3.4*  AF271080 / U61753 | 85 | Fwd: ATTGGCCTGTCCAAAACACA  Rev: AGCTTCAGATCAAGGAAGAAGTTC | TGGAATCTGTGTGTCTGAACCCC | [5] | chr17:18017732 to 18017796  Chr13: 22193859 to 22193923 | NC_035093  NC_035089 |
| *cathelicidin 1a*  AY382478 | 189 | Fwd: TCTCTCGTCCTGGGGTT  Rev: GTTGTAGCGTGCTGATCTATG | TAATTGGTCGTCCTGGGGGTGG | [3] | chr28:12847647 to 12847835 | NC_035104 |
| *cathelicidin 2a*  AY360356 | 135 | Fwd: AAAGATTCCAAGGGGGGT  Rev: CAAAGGGTGTGTTGTGCTGT | GCTCTCGTCCTGGGTTTGGCTCC | [6] | chr8:67206588 to 67206722 | NC_035084 |
| *ifn γ1* and *ifn γ2*  FJ184374 / FJ184375 | 68 | Fwd: AAGGGCTGTGATGTGTTTCTG  Rev: TGTACTGAGCGGCATTACTCC | TTGATGGGCTGGATGACTTTAGGA | [7] | unplaced genomic scaffold:1160 to 1227 | NW_018573689 |
| *igdm*  AY870262 | 304 | Fwd: CAGGAGGAAAGTTCGGCATCA  Rev: CCTCAAGGAGCTCTGGTTTGGA | CCACACCACACAGACTCTGGCCCTGAA | [8] | chr:1348059335 to 48059646 48383504 | NC_035089 |
| *igds*  JQ003979 | 304 | Fwd: TGGCACGCCAGGATTTGAC  Rev: TCAGAATTGAGTGAACGGACAGACA | CCACACCACACAGACTCTGGCCCTGAA | [8] | chr13:48059371 to 48059486 | NC_035089 |
| ^MS^ *igm*  S63348 / AH014877 | 72 | Fwd: ACCCTCCTCTTGGTCGTTTC  Rev: TGATGACACCAACAGCAACA | TGATGACACCAACAGCAACA | [7] | chr12:81780295 to 81780366 | NC_035088 |
| ^MS^ *igt*  AY870265 / AY870263 | 73 | Fwd: AGCACCAGGGTGAAACCA  Rev: GCGGTGGGTTCAGAGTCA | AGCAAGACGACCTCCAAAACAGAAC | [7] | chr12:81681064 to 81681095 chr13:48285298 to 48285339 | NC_035088 NC_035089 |
| *il-1β*  AJ223954 | 91 | Fwd: ACATTGCCAACCTCATCATCG  Rev: TTGAGCAGGTCCTTGTCCTTG | CATGGAGAGGTTAAAGGGTGGC | [7] | chr6:42311100 to 42311190 | NC_035082 |
| *il-2a*  FJ571513 | 110 | Fwd: ATGCAACACCACATCAGCAT  Rev: TGCCACGGCCCTACAAAAGA  RE  TGCCACGGCCCTACAAAAGA | TGCCACGGCCCTACAAAAGA | [3] | chr2:30110175 to 30110224 | NC_035101 |
| *il-4/13a*  AB574337 | 138 | Fwd: ATCCTTCTCCTCTCTGTTGC  Rev: GAGTGTGTGTGTATTGTCCTG | CGCACCGGCAGCATAGAAGT | [9] | chr12:50133310 to 50133448 | NC_035088 |
| *il-6a*  DQ866150 | 91 | Fwd: ACTCCCCTCTGTCACACACC  Rev: GGCAGACAGGTCCTCCACTA | CCACTGTGCTGATAGGGCTGG | [10] | chr14:7102251 to 7102305 | NC_035090 |
| *il-8* isoforms *a, b , c, d* & *e*  AY160982 to AY160986 | 69 | Fwd: AGAATGTCAGCCAGCCTTGT  Rev: TCTCAGACTCATCCCCTCAGT | TTGTGCTCCTGGCCCTCCTGA | [10] | chr12:74891818 to 74891878 chr12:74911280 to 74911340 chr:13:41793683 to 41793743 | NC_035088 NC_035088 NC_035089 |
| *il-10a*  [AB118099](http://www.ncbi.nlm.nih.gov/entrez/viewer.fcgi?db=nucleotide&val=47678892) | 70 | Fwd: CGACTTTAAATCTCCCATCGAC  Rev: GCATTGGACGATCTCTTTCTTC | CATCGGAAACATCTTCCACGAGCT | [7] | chr7:77880575 to 77880746 | NC_035083 |
| ^1^ *il-12 α* chain  HE798148 | 84 | Fwd: CAACGGAACACCACATTCAG  Rev: AGCCTGTAGTGAGGCAGCAT | TGCGTGTCTGAGGAACATCCG | [11] | chr27:15869014 to 15869075 | NC_035103 |
| *il-17a/f2a*  AJ580842 | 158 | Fwd: TCAAAAGCAACGTGTCGAAG  Rev: TCCCTCTGATTCCTCTGTGG | TATGCTGCTGGGCCTGACCA | [11] | chr19:32910058 to 32910190 | NC_035095 |
| *il-17c1*  CAW30792 | 138 | Fwd: CTGGCGGTACAGCATCGATA  Rev: GAGTTATATCCATAATCTTCGTATTCGGC | CGTGATGTCCGTGCCCTTTGACGATG | [9] | chr1:22090819 to 22090948 | NC_035077 |
| *il-17c2*  CAW30793 | 134 | Fwd: CTGGCGGTACAGCATCGATA  Rev: CAGAGTTATATGCATGATGTTGGGC | CGTGGTGTCCAGGCCCTTTAATGATG | [9] | chr2:51653897 to 51654020 | NC_035078 |
| *il-22*  AM748537 | 64 | Fwd: ATGACCACCACCACAGCATT  Rev: ATTCCTTTCCCCTCCTCCAT | CTTTCCGCAAGAAGTTGTCCGAG | [12] | chr13:50267165 to 50267208 | NC_035089 |
| *lysozyme cii*  X59491 | 188 | Fwd: GAAACAGCCTGCCCAACT  Rev: GTCCAACACCACACGCTT | ATACCCAGGCCACCAACCGCAACAC | [13] | chr5:6936784 to 6936945 | NC_035081 |
| *saa*  AM422446 | 79 | Fwd: GGGAGATGATTCAGGGTTCCA  Rev: TTACGTCCCCAGTGGTTAGC | TCGAGGACACGAGGACTCAGCA | [14] | chr6:67564044 to 67564107 | NC_035082 |
| *tcr-β*  AF329700 | 73 | Fwd: TCACCAGCAGACTGAGAGTCC  Rev: AAGCTGACAATGCAGGTGAATC | CCAATGAATGGCACAAACCAGAGAA | [7] | chr25:82009983 to 82010055 | NC_035101 |
| *tgf-β1a*  [X99303](http://www.ncbi.nlm.nih.gov/entrez/viewer.fcgi?db=nucleotide&val=1478246) | 75 | Fwd: TCTGAATGAGTGGCTGCAAG  Rev: GGTTTCCCACAATCACAAGG | CTGGAGAGGAGCAGGGATTCCAAT | [7] | chr29:30680564 to 30681150 | NC_035105 |
| *tnf-α1* & *tnf-α2*  AJ277604 / AJ401377 | 75 | Fwd: GGGGACAAACTGTGGACTGA  Rev: GAAGTTCTTGCCCTGCTCTG | GACCAATCGACTGACCGACGTGGA | [10] | chr3:29836438 to 29836503 chr2:19874096 to 19874161 | NC_035079 NC_035078 |
| *aopo* (*A salmonicida*)  DQ386862 | 248 | Fwd: AGCTCATCCAATGTTCGGTATT  Rev: AAGTTCATCG TGCTGTTCCA | GGCGCTCAATCCGGTTTACCCACGG | [15] | Not relevant | Not relevant |

^R^ reference genes (housekeeping genes).

^MS^ qPCR assay targets both membrane bound and secreted forms.

^1^ The α chain of IL-12 is in common to two isoforms of IL-12.

1. Purcell, M.K., et al., *Quantitative expression profiling of immune response genes in rainbow trout following infectious haematopoietic necrosis virus (IHNV) infection or DNA vaccination.* Fish & Shellfish Immunology, 2004. **17**(5): p. 447-462.

2. Sugiura, S.H., K. Kelsey, and R.P. Ferraris, *Molecular and conventional responses of large rainbow trout to dietary phosphorus restriction.* J Comp Physiol B, 2007. **177**(4): p. 461-72.

3. Marana, M.H., et al., *Subunit vaccine candidates against Aeromonas salmonicida in rainbow trout Oncorhynchus mykiss.* PLOS ONE, 2017. **12**(2): p. e0171944.

4. Ingerslev, H.-C., et al., *Expression profiling and validation of reference gene candidates in immune relevant tissues and cells from Atlantic salmon (Salmo salar L.).* Molecular Immunology, 2006. **43**(8): p. 1194-1201.

5. Raida, M.K. and K. Buchmann, *Innate immune response in rainbow trout (Oncorhynchus mykiss) against primary and secondary infections with Yersinia ruckeri O1.* Dev Comp Immunol, 2009. **33**(1): p. 35-45.

6. Xueqin, J., P.W. Kania, and K. Buchmann, *Comparative effects of four feed types on white spot disease susceptibility and skin immune parameters in rainbow trout, Oncorhynchus mykiss (Walbaum).* Journal of Fish Diseases, 2012. **35**(2): p. 127-135.

7. Raida, M.K. and K. Buchmann, *Temperature-dependent expression of immune-relevant genes in rainbow trout following Yersinia ruckeri vaccination.* Diseases of Aquatic Organisms, 2007. **77**(1): p. 41-52.

8. Skov, J., et al., *Effects of soluble immunostimulants on mucosal immune responses in rainbow trout immersion-vaccinated against Yersinia ruckeri.* Aquaculture, 2018. **492**: p. 237-246.

9. Chettri, J.K., et al., *Epidermal response of rainbow trout to Ichthyobodo necator: immunohistochemical and gene expression studies indicate a Th1-/Th2-like switch.* J Fish Dis, 2014. **37**(9): p. 771-83.

10. Raida, M.K. and K. Buchmann, *Bath vaccination of rainbow trout (Oncorhynchus mykiss Walbaum) against Yersinia ruckeri: effects of temperature on protection and gene expression.* Vaccine, 2008. **26**(8): p. 1050-62.

11. Jaafar, R.M., et al., *Effects of adjuvant Montanide™ ISA 763 A VG in rainbow trout injection vaccinated against Yersinia ruckeri.* Fish & Shellfish Immunology, 2015. **47**(2): p. 797-806.

12. Olsen, M.M., et al., *Cellular and humoral factors involved in the response of rainbow trout gills to Ichthyophthirius multifiliis infections: Molecular and immunohistochemical studies.* Fish & Shellfish Immunology, 2011. **30**(3): p. 859-869.

13. Chettri, J.K., et al., *Differential immune response of rainbow trout (Oncorhynchus mykiss) at early developmental stages (larvae and fry) against the bacterial pathogen Yersinia ruckeri.* Developmental & Comparative Immunology, 2012. **36**(2): p. 463-474.

14. Skov, J., et al., *Immunomodulatory effects of dietary beta-1,3-glucan from Euglena gracilis in rainbow trout (Oncorhynchus mykiss) immersion vaccinated against Yersinia ruckeri.* Fish Shellfish Immunol, 2012. **33**(1): p. 111-20.

15. Fernandez-Alvarez, C., S.F. Gonzalez, and Y. Santos, *Development of a SYBR green I real-time PCR assay for specific identification of the fish pathogen Aeromonas salmonicida subspecies salmonicida.* Appl Microbiol Biotechnol, 2016. **100**(24): p. 10585-10595.
